# The application of probabilistic linguistic CODAS method based on new score function in multi-criteria decision-making

**DOI:** 10.1007/s40314-021-01568-6

**Published:** 2021-12-13

**Authors:** Liuxin Chen, Xiaoling Gou

**Affiliations:** Laboratory of Intelligent Analysis and Decision on Complex Systems, Chongqing University of Posts and Telecommunications, Chongqing, 400065 People’s Republic of China

**Keywords:** Probabilistic linguistic term sets (PLTSs), Multi-criteria decision-making (MCDM), Score function, PL-CODAS method, 93-08

## Abstract

Probabilistic linguistic term sets (PLTSs) play an important role in multi-criteria decision-making(MCDM) problems because it can not only describe objects with several possible linguistic terms, but also represent the proportion of each linguistic term, which can effectively avoid the distortion of decision information to a greater extent and ensure the credibility of decision results. First, to compare PLTS more simply and reasonably, we define a new score function that takes into account partial deviations. Then considering the superiority of the classic combinative distance-based assessment (CODAS) method in the complete representation of information, it is extended to the probabilistic linguistic environment. Subsequently, we improved the classic CODAS method and proposed the PL-CODAS method. Finally, we apply the PL-CODAS method to a cases of venture investors choosing emerging companies, and we compare the proposed method with PL-TOPSIS method, PL-TODIM method and PL-MABAC method to verify its applicability and effectiveness.

## Introduction

When evaluate the real things, the accurate data are difficult to obtain or the cost is too high to achieve this goal and the decision-makers (DMs) may prefer to assess the objects using the linguistic terms rather than the numerical values (Bonissone 1980; Zhan 2002; Liu et al. 2104; Wang et al. 2014). For example, when we evaluate a person’s appearance, we have no way to express it quantitatively, and often use linguistic terms such as very good looking, good looking, general and bad looking.

In this case, Zadeh (1975) put forward the fuzzy linguistic approach(FLA) to model the qualitative linguistic assessment information. Subsequently, the combination of linguistic information and existing uncertain sets was widely used. Yager (1981) introduced the ordinal scale-based model. Degani and Bortolan (1988) introduced the linguistic approximation method. Bordogna and Pasi (1993)combined fuzzy linguistic approach with Boolean information and introduced a formal extended linguistic model. Herrera and Martinez (2000) combined type 2 fuzzy sets with linguistic terms, allowing linguistic terms to be expressed continuously on a regional basis. Xu (2004) defined the basic aggregation operation of linguistic terms and proposed the virtual linguistic model. Although linguistic term has been expanded to a large extent, these studies still have certain limitations, especially on linguistic information modeling. The reason is that they can only provide a single linguistic term to express the linguistic evaluation information of decision-makers about linguistic variables. Decision-makers are often not very clear when making evaluations, and may hesitate between several evaluation values. To describe this evaluation, Rodriguez et al. (2012) put forward the concept of hesitant fuzzy linguistic term set (HFLTS), which is a combination of hesitant fuzzy sets (HFS) (Torra 2010) and fuzzy linguistic approach (FLA) (Zadeh 1975). It allows decision-makers to describe an object in multiple linguistic terms simultaneously. However, each of linguistic terms is considered equal probability for HFLTS, which is inconsistent with some situations in practice. For example, let five consumers give feedback on the product, four of them are satisfied and one is dissatisfied. Under the HFLTS, the evaluation information is denoted as: $\{$satisfied, dissatisfied$\}$. Obviously, it is unreasonable not to consider the importance of terms in different linguistic. Based on the above considerations, Pang et al. (2016) first proposed the probabilistic linguistic term set (PLTS), which can reduce the information loss in the evaluation process by giving each linguistic term a probability to denote its importance degree or weight.

As for the comparisons of PLTSs, Pang et al. (2016) defined the score function when they proposed PLTS. They compare PLTS with the mean of linguistic terms, and use their variance to compare if the means are equal. Subsequently, a large number of scholars studied the possibility on PLTSs (Zhao et al. 2018; Xian et al. 2019; Feng et al. 2019; Yu et al. 2019; Liu and Teng 2019). Bai et al. (2017) proposed a diagram method to analyze the structures of PLTSs and put forward a possibility degree formula for comparing PLTSs. In 2019, Yu et al. (2019) proposed a new comparison rule of PLTSs, which compared each linguistic term one by one. For the score function proposed in literature (Pang et al. 2016), the deviation of the term set is considered if and only if the mean is equal, and it does not take into account the hesitation of PLTSs. Therefore, there is the deficiency of missing information. The comparison rules proposed by Bai et al. (2017) and Yu et al. (2019) are more complicated and the amount of calculation is relatively large, so it is very necessary to find a comparison rule that has a smaller amount of calculation and can retain as much information as possible.

Since Markowitz proposed the portfolio selection theory based on variance, the variance (mean variance) has become a very influential classic financial risk measure, which is called the volatility method. The most important features of the variance calculation is simple and has a fairly mature theory. However, this method also accounts the deviations above the mean when calculating fluctuations, which is unreasonable. Based on this idea, we propose to consider only the deviation of the linguistic terms value below the mean, i.e. partial variance. Subsequently, we define a comprehensive score function on PLTS by the concept of partial variance, which not only considers the mean and hesitation of linguistic terms, but also considers partial variance.

The CODAS (Ghorabaee et al. 2016) method was first proposed by Ghorabaee et al. in 2016. In this decision-making method, two distance measures, Euclidean distance and Hamming distance, are used to determine the desirability of alternatives by the distance from the negative ideal solution. Since the CODAS method was proposed, it has been widely used in various uncertain sets (Bolturk 2018; Yalcin and Pehlivan 2019; Villalpando et al. 2019; Maghsoodi et al. 2020; Gndogdu and Kahraman 2019; Seker 2020; Tuysuz and Kahraman 2020). Ghorabaee et al. (2017) combined linguistic variables and trapezoidal fuzzy numbers with the CODAS method, and applied the extension method to a numerical example of a shoe company. Pamucar et al. (2018) used the linguistic neutral number to modify the CODAS method, and proposed a new LNN PW-CODAS model, and finally applied it to the selection of the optimal power generation technology. Panchal et al. (2017) combined AHP with CODAS method and introduced the fuzzy AHP-CODAS framework. Badi et al. (2018) applied CODAS method to the problem of sea water desalination site selection. He et al. (2019) combined CODAS method with Pythagorean 2-tuple linguistic information. However, among the above-mentioned researches, there is less research on the CODAS method under probabilistic linguistic environments. The goal of this paper is to extend the CODAS method to solve the probabilistic linguistic MCDM with unknown weight information. The innovation of the paper can be summarized as follows:

1. In the case of risk assessment, define a score function on the probabilistic linguistic term set, which comprehensively considers the mean and partial variance. The score function can achieves one-step comparison of probabilistic linguistic term set.
2. Put forward an extended probabilistic linguistic CODAS (PL-CODAS) approach to handle MCDM problems where the criteria weights on the probabilistic linguistic term set are completely unknown.
3. Utilize the extended PL-CODAS method to a cases of venture investors choosing emerging companies.
4. Compare with the three existing methods to demonstrate the rationality and availability of the proposed approach.

To achieve this goal, the rest of this paper is organized as follows. Section 2 reviews basic concepts and existing studies of PLTSs briefly. Section 3 defines a new comparison rule on PLTS and gives the calculation formula of the score function. In Sect. 4, the shortcomings of CODAS method are proposed, and then the original method is improved. In Sect. 5, the decision method of the CODAS method on PLTSs is constructed and detailed steps are given. In Sect. 6, a decision-making problem about the choice of emerging enterprises in venture capital is given to show the practicality of the improved CODAS method under probabilistic linguistic environment. Subsequently, some comparisons between the improved CODAS method and other existing methods are made to illustrates the effectiveness of improved CODAS method. Finally, the paper is summarized in Sect. 7.

## Preliminaries

In this section, we introduce some basic concepts, such as linguistic scale functions, probabilistic linguistic term set (PLTS) and so on.

### The linguistic term set

Suppose that $S = \{ {s_t}|t = 0,1, \ldots , l - 1\}$ be a linguistic term set (LTS) with odd cardinality, where ${s_t}$ represents possible value for a linguistic variable. In addition, the linguistic elements in *S* should satisfy the following conditions (Rodriguez et al. 2012):

1. If ${s_a},{s_b} \in S$ and $a < b$, then ${s_a} < {s_b}$;
2. There exists the negation operator: $neg({s_a}) = {s_b}$, where $a = l \mathrm{{ - }}1\mathrm{{ - b}}$;
3. $$\mathrm{Max} ({s_a},{s_b}) = {s_a}$$, if $${s_a} \ge {s_b}$$;
4. $$\mathrm{Min} ({s_a},{s_b}) = {s_a}$$, if $${s_a} \le {s_b}$$.

For example, when $l = 7$, then *S* could be given as follows: $S\! =\! \{ {s_0} \!=\! \mathrm{extremely} \ \mathrm{poor}, {s_1} = \mathrm{poor}, {s_2} = \mathrm{slight} \ \mathrm{poor}, {s_3} = \mathrm{fair}, {s_4} = \mathrm{slight} \ \mathrm{good},{s_5} = \mathrm{good}, {s_6} = \mathrm{very} \ \mathrm{good}\}$.

Furthermore, to retain original decision information as much as possible, Dai et al. (2008) extended the discrete linguistic term set to a continuous form ${\overline{S}} = \{ {s_t}|t \in [0,l-1]\}$, where *l* is a sufficiently large positive integer.

### Linguistic scale functions

Traditional operational laws of fuzzy linguistic approach are to calculate directly by utilizing the subscript of LT based on an assumption that the deviations between adjacent linguistic terms are equal. It does not completely match the actual situation. For instance, the DM may believe that the deviation between "fair" and "slight good" is less than the deviation between "slight good" and "good" in terms of the risk level of the stock market. In addition, the result of the symbolic operation may exceed the bounder limit of predefine linguistic term set, which seems unreasonable. Committed to solving such problems, Wang et al. (2014) proposed the concept of linguistic scale functions (LSFs).

#### Definition 1

(Wang et al. 2014). Let $S = \left\{ {{s_0},{s_1}, \ldots ,{s_{2\tau }}} \right\}$ be a LTS and ${\theta _i}$ be a numeric value which represents the semantic of $s_i$. Then the linguistic scale functions (LSFs) *f* is the mapping from $s_i$ to ${\theta _i}(i = 0,1, \ldots , 2\tau )$ is defined as

1 $$\begin{aligned} f:{s_i} \rightarrow {\theta _i}(i = 0,1, \ldots , 2\tau ). \end{aligned}$$

When the deviations between adjacent linguistic terms are increasing with the extension from $s_\tau$:

2 $$\begin{aligned} f\left( {{s_i}} \right) = {\theta _i} = \left\{ \begin{array}{*{20}{l}} \frac{{{a^\tau } - {a^{\tau - i}}}}{{2{a^\tau } - 2}}, &{}\quad i = 0,1, \ldots ,\tau ;\\ \frac{{{a^\tau } + {a^{i - \tau }} - 2}}{{2{a^\tau } - 2}}, &{}\quad i = \tau + 1,\tau + 2, \ldots ,2\tau . \end{array} \right. \end{aligned}$$

Obviously, there exists a function ${f^{ - 1}}$ such that numeric value can be translated into linguistic term as follows:

3 $$\begin{aligned} {f}^{ - 1}\left( {{\theta _i} } \right) = s_i=\left\{ {\begin{array}{*{20}{l}} {s_{\tau - {{\log }_a}({a^\tau } - (2{a^\tau } - 2){\theta _i} )}},&{}\quad {\theta _i} \in \left[ {0,\frac{1}{2}} \right] ;\\ {s_{\tau + {{\log }_a}((2{a^\tau } - 2){\theta _i} - {a^\tau } + 2)}},&{}\quad {\theta _i} \in \left( {\frac{1}{2},1} \right] , \end{array}} \right. \end{aligned}$$

where the parameter *a* represents the degree of preference, which is subjectively determined by DM according to the actual situation. Assume that indicator *P* is far more important than indicator *Q*, and the importance ratio is *m*, then ${a^k} = m$ (*k* represents the scale level). The current academic community generally believes that the importance ratio is 9, then we can obtain $a = \root k \of {9}$. For example, when the given linguistic term set is $S = \left\{ {{s_0},{s_1}, \ldots ,{s_6}} \right\}$, the scale level is 7, then $a = \root 7 \of {9} \approx 1.37$.

### Probabilistic linguistic term set

When expressing preference information, considering that DM may hesitate between several linguistic terms that are unequal importance, Pang et al. (2016) presented the following concept of probabilistic linguistic term set(PLTS).

#### Definition 2

(Pang et al. 2016). Let $S = \left\{ {{s_0},{s_1}, \ldots ,{s_{2\tau }}} \right\}$ be a linguistic term set, a PLTS can be defined as:

4 $$\begin{aligned} L\left( p \right) = \left\{ {{L^{\left( k \right) }}\left( {{p^{\left( k \right) }}} \right) \left| {{L^{\left( k \right) }} \in S,{p^{\left( k \right) }} \ge 0,k = 1,2, \ldots ,\# L\left( p \right) ,\sum \limits _{k = 1}^{\# L\left( p \right) } {{p^{\left( k \right) }} \le 1} } \right. } \right\} . \end{aligned}$$

where ${L^{\left( k \right) }}\left( {{p^{\left( k \right) }}} \right)$ is the linguistic term ${L^{\left( k \right) }}$ associated with the probability ${p^{\left( k \right) }}$, and $\# L\left( p \right)$ is the number of all different linguistic terms in $L\left( p \right)$.

It is worth noting that if $\sum \nolimits _{k = 1}^{\# L\left( p \right) } {{p^{\left( k \right) }} = 1}$, then we have the complete information of probability distribution of all possible linguistic terms; if $\sum \nolimits _{k = 1}^{\# L\left( p \right) } {{p^{\left( k \right) }} < 1}$, there exist some partial ignorance because people’s current knowledge is not enough to provide complete assessment information, which is the most common situation in practical decision problems. Especially, if $\sum \nolimits _{k = 1}^{\# L\left( p \right) } {{p^{\left( k \right) }} = 0}$, it means completely ignorance.

#### Definition 3

(Gou and Xu 2016). Let ${L_i}\left( p \right) = \left\{ {L_i^{\left( {{k_i}} \right) }\left( {p_i^{\left( {{k_i}} \right) }} \right) \left| {{k_i} = 1,2, \ldots ,\# {L_i}\left( p \right) } \right. } \right\} (i=1,2)$ be any two PLTS and $\lambda > 0$, then the operational rules of PLTS are defined as follows:

1. $${L_1}\left( p \right) \oplus {L_2}\left( p \right) = { \cup _{L_1^{\left( {{k_1}} \right) } \in {L_1}\left( p \right) ,L_2^{\left( {{k_2}} \right) } \in {L_2}\left( p \right) }} \Big \{ {f^{ - 1}}\Big ( {f\left( {L_1^{\left( {{k_1}} \right) }} \right) + f\left( {L_2^{\left( {{k_2}} \right) }} \right) - f\left( {L_1^{\left( {{k_1}} \right) }} \right) f\left( {L_2^{\left( {{k_2}} \right) }} \right) } \Big )\left( {p_1^{\left( {{k_1}} \right) }p_2^{\left( {{k_2}} \right) }} \right) \Big \}; $$
2. $${L_1}\left( p \right) \otimes {L_2}\left( p \right) = { \cup _{L_1^{\left( {{k_1}} \right) } \in {L_1}\left( p \right) ,L_2^{\left( {{k_2}} \right) } \in {L_2}\left( p \right) }}\left\{ {{f^{ - 1}}\left( {f\left( {L_1^{\left( {{k_1}} \right) }} \right) f\left( {L_2^{\left( {{k_2}} \right) }} \right) } \right) \left( {p_1^{\left( {{k_1}} \right) }p_2^{\left( {{k_2}} \right) }} \right) } \right\} $$;
3. $$\lambda {L_1}\left( p \right) = { \cup _{{L_1}^{\left( {{k_1}} \right) } \in {L_1}\left( p \right) }}\left\{ {{f^{ - 1}}\left( {1 - {{\left( {1 - f\left( {{L_1}^{\left( {{k_1}} \right) }} \right) } \right) }^\lambda }} \right) \left( {{p_1}^{\left( {{k_1}} \right) }} \right) } \right\} ;$$
4. $${\left( {{L_1}\left( p \right) } \right) ^\lambda } = { \cup _{{L_1}^{\left( {{k_1}} \right) } \in {L_1}\left( p \right) }}\left\{ {{f^{ - 1}}\left( {{{\left( {f\left( {{L_1}^{\left( {{k_1}} \right) }} \right) } \right) }^\lambda }} \right) \left( {{p_1}^{\left( {{k_1}} \right) }} \right) } \right\} $$;
5. $$neg\left( {{L_1}\left( p \right) } \right) = \left\{ {\bar{L}_1^{\left( {{k_1}} \right) }\left( {p_1^{\left( {{k_1}} \right) }} \right) \left| {{\bar{L}}_1^{\left( {{k_1}} \right) } = neg\left( {L_1^{\left( {{k_1}} \right) }} \right) ,{k_1} = 1,2, \ldots ,\# {L_1}\left( p \right) } \right. } \right\} $$.

Since the position of the elements in the set can be swapped arbitrarily, for the sake of calculation, the PLTS are ranked in descending order of ${L^{\left( k \right) }}{p^{\left( k \right) }}$ $\left( {k = 1,2, \ldots ,\# L\left( p \right) } \right)$. There are two distinct tasks in the normalization of PLTSs. The first one is to estimate the ignorance of probabilistic information, and the second one is to normalize the cardinality of a PLTS for the purpose of computation.

#### Normalization of PLTS

The normalization process is crucial in decision-making problems, and there are two distinct tasks in the normalization of PLTS. Eliminate the ignorance of probabilistic information and normalize the cardinality of a PLTS.

##### Definition 4

(Pang et al. 2016). Given a PLTS $L\left( p \right)$ with $\sum \nolimits _{k = 1}^{\# L\left( p \right) } {{p^{\left( k \right) }} < 1}$, then the associated PLTS $\dot{L}\left( p \right)$ is defined by

5 $$\begin{aligned} \dot{L}\left( p \right) = \left\{ {{L^{\left( k \right) }}\left( {{{\dot{p}}^{\left( k \right) }}} \right) \left| {k = 1,2, \ldots ,\# L\left( p \right) } \right. } \right\} . \end{aligned}$$

where ${\dot{p}^{\left( k \right) }} = {p^{\left( k \right) }}/\sum \nolimits _{k = 1}^{\# L\left( p \right) } {{p^{\left( k \right) }}}$, for all $k = 1,2, \ldots ,\# L\left( p \right)$.

##### Definition 5

(Pang et al. 2016). Let $$ {L_i}\left( p \right) = \left\{ {L_i^{\left( {{k_i}} \right) }\left( {p_i^{\left( {{k_i}} \right) }} \right) \left| {{k_i} = 1,2, \ldots ,\# {L_i}\left( p \right) } \right. } \right\} (i=1,2)$$ be any two PLTSs and $$\# {L_1}\left( p \right) $$ and $$\# {L_2}\left( p \right) $$ be the numbers of linguistic terms in $${L_1}\left( p \right) $$ and $${L_2}\left( p \right) $$, respectively. If $$\# {L_1}\left( p \right) $$ > $$\# {L_2}\left( p \right) $$, then we add $$\# {L_1}\left( p \right) $$ $$- $$ $$\# {L_2}\left( p \right) $$ linguistic terms to $${L_2}\left( p \right) $$, so that the numbers of linguistic terms in $${L_1}\left( p \right) $$ and $${L_2}\left( p \right) $$ are identical. The added linguistic terms are the smallest ones in $${L_2}\left( p \right) $$, and the probability of all added linguistic terms are zero.

Based on the discussion above, the exact normalization process can be summarized as the following two steps:

1. If $\sum \nolimits _{k = 1}^{\# L\left( p \right) } {{p^{\left( k \right) }} < 1}$, by Definition 4, we can calculate ${\dot{L}_i}\left( p \right) (i = 1,2$).
2. If $\# {L_1}\left( p \right)$ $\ne$ $\# {L_2}\left( p \right)$, then according to Definition 5, we add some elements to shorter collections. For convenience, the normalized PLTS are still denoted by ${L}\left( p \right)$.

#### Distance measure of PLTS

Distance measure is an effective tool for dealing with DM problems in the context of fuzzy linguistic information, which can visually depict the differences between things. In this part, we review several distance measures between PLTS.

##### Definition 6

(Lin and Xu 2018). Let $L_1^{\left( {{k_1}} \right) }\left( {p_1^{\left( {{k_1}} \right) }} \right) \in {L_1}\left( p \right)$ and $L_2^{\left( {{k_2}} \right) }\left( {p_2^{\left( {{k_2}} \right) }} \right) \in {L_2}\left( p \right)$ be any two PLTSs, then the distance measure between them is defined as

6 $$\begin{aligned} d\left( {L_1^{\left( {{k_1}} \right) }\left( {p_1^{\left( {{k_1}} \right) }} \right) ,L_2^{\left( {{k_2}} \right) }\left( {p_2^{\left( {{k_2}} \right) }} \right) } \right) = \left| {p_1^{\left( {{k_1}} \right) }f\left( {L_1^{\left( {{k_1}} \right) }} \right) - p_2^{\left( {{k_2}} \right) }f\left( {L_2^{\left( {{k_2}} \right) }} \right) } \right| , \end{aligned}$$

where $f\left( {L_1^{\left( {{k_1}} \right) }} \right)$ and $f\left( {L_2^{\left( {{k_2}} \right) }} \right)$ are calculated by Eq. (2).

Given two PLTSs ${L_1}\left( p \right)$ and ${L_2}\left( p \right)$, according to Definition 6, the normalized hamming distance and normalized Euclidean distance of ${L_1}\left( p \right)$ and ${L_2}\left( p \right)$ are, respectively, shown as follows:

7 $$\begin{aligned} {d_{nh}}\left( {{L_1}\left( p \right) ,{L_2}\left( p \right) } \right) = \frac{1}{{\# {L_1}\left( p \right) }}\sum \limits _{k = 1}^{\# {L_1}\left( p \right) } {d\left( {L_1^{\left( k \right) }\left( {p_1^{\left( k \right) }} \right) ,L_2^{\left( k \right) }\left( {p_2^{\left( k \right) }} \right) } \right) }, \end{aligned}$$

8 $$\begin{aligned} {d_{ne}}\left( {{L_1}\left( p \right) ,{L_2}\left( p \right) } \right) = \sqrt{\frac{1}{{\# {L_1}\left( p \right) }}\sum \limits _{k = 1}^{\# {L_1}\left( p \right) } {{{\left( {d\left( {L_1^{\left( k \right) }\left( {p_1^{\left( k \right) }} \right) ,L_2^{\left( k \right) }\left( {p_2^{\left( k \right) }} \right) } \right) } \right) }^2}} }, \end{aligned}$$

where $${L_1}\left( p \right) $$ and $${L_2}\left( p \right) $$ are normalized form through Definitions 4 and 5; thus, $$\# {L_1}\left( p \right) $$ $$= $$ $$\# {L_2}\left( p \right) $$.

## A novel score function of PLTS

After the information is represented in the form of probabilistic linguistic variables, how to effectively compare them is a very important issue in the decision-making process. The most common comparison way is based on their score function, and aroused great enthusiasm for scholars. Pang et al. (2016) defined the score function of PLTS as $E\left( {L\left( p \right) } \right) = {S_{{\bar{\alpha }} }} = \sum \nolimits _{k = 1}^{\# L\left( p \right) } {{r^{\left( k \right) }}{p^{\left( k \right) }}/} \sum \nolimits _{k = 1}^{\# L\left( p \right) } {{p^{\left( k \right) }}}$ ${{r^{\left( k \right) }}}$ based on the subscript of linguistic term ${{L^{\left( k \right) }}}$. Essentially, the score value is expressed by the mean value. When the mean of two PLTS is the same, the variance between them will further distinguish them. The smaller the variance, the more concentrated the data, and the better the result. For the above comparison rules, there still exist the following deficiencies:

1. The score function only considers the mean of PLTS but not reflect the hesitation of them.
2. Variance can be considered if and only if the mean is the same. However, in practical applications, people are more willing to through a comprehensive comparison method to reflect the differences of things.

Based on the above problems, Shen et al. (2019) uses the mean minus the relative variance to define the score function, and gives a comprehensive score function formula. Although it takes into account the linguistic terms and the deviation between linguistic terms of PLTS simultaneously, there are still the following two shortcomings:

1. Subtracting the relative variance from the mean is a meaningless quantity, and it is not very accurate to describe the score function.
2. When calculating the score function with variance, it is believed that a deviation greater than the mean will also reduce the value of the score function, which is contrary to our actual life experience. We generally think that only deviations less than the mean will affect the score, and deviations greater than the mean should not reduce the score.

Based on above analysis, we only calculate the variance below the mean when describing the volatility (i.e. risk) of the evaluation values and propose the novel score function of PLTS.

### Definition 7

Let ${L}\left( p \right) = \left\{ {L^{\left( {{k}} \right) }\left( {p^{\left( {{k}} \right) }} \right) \left| {k = 1,2, \ldots ,\# {L}\left( p \right) } \right. } \right\}$ be a PLTS, where $S = \left\{ {{s_0},{s_1}, \ldots ,{s_{2\tau }}} \right\}$. Then the mean and partial variance of $L\left( p \right)$ are defined as follows:

9 $$\begin{aligned}&E\left( {L\left( p \right) } \right) = \sum \nolimits _{k = 1}^{\# L\left( p \right) } {{p^{\left( k \right) }}f\left( {{L^{\left( k \right) }}} \right) } /\sum \nolimits _{k = 1}^{\# L\left( p \right) } {{p^{\left( k \right) }}}, \end{aligned}$$

10 $$\begin{aligned}&PVar\left( {L\left( p \right) } \right) = \sum \nolimits _{k = 1}^{\# L\left( p \right) } {\Gamma \left( {f\left( {{L^{\left( k \right) }}} \right) } \right) {p^{\left( k \right) }}{{\left( {f\left( {{L^{\left( k \right) }}} \right) - E\left( {L\left( p \right) } \right) } \right) }^2}} /\sum \nolimits _{k = 1}^{\# L\left( p \right) }\nonumber \\&{\Gamma \left( {f\left( {{L^{\left( k \right) }}} \right) } \right) {p^{\left( k \right) }}}, \end{aligned}$$

where ${f\left( {{L^{\left( k \right) }}} \right) }$ calculated by Eq. (2), and ${\Gamma \left( {f\left( {{L^{\left( k \right) }}} \right) } \right) }$ denotes a threshold function to identify the value relationship between ${f\left( {{L^{\left( k \right) }}} \right) }$ and ${E\left( {L\left( p \right) } \right) }$, and defined as follows:

11 $$\begin{aligned} \Gamma \left( {f\left( {{L^{\left( k \right) }}} \right) } \right) = \left\{ {\begin{array}{*{20}{l}} {\begin{array}{*{20}{c}} {1,}&{}\quad {f\left( {{L^{\left( k \right) }}} \right) < E\left( {L\left( p \right) } \right) }; \end{array}}\\ {\begin{array}{*{20}{l}} {0,}&{}\quad {f\left( {{L^{\left( k \right) }}} \right) \ge E\left( {L\left( p \right) } \right) }. \end{array}} \end{array}} \right. \end{aligned}$$

### Definition 8

Let ${L}\left( p \right) = \left\{ {L^{\left( {{k}} \right) }\left( {p^{\left( {{k}} \right) }} \right) \left| {k = 1,2, \ldots ,\# {L}\left( p \right) } \right. } \right\}$ be a PLTS, where $S = \left\{ {{s_0},{s_1}, \ldots ,{s_{2\tau }}} \right\}$. Then the score function of $L\left( p \right)$ is

12 $$\begin{aligned} SF\left( {L\left( p \right) } \right) = E\left( {L\left( p \right) } \right) \left( {1 - \frac{{PVar\left( {L\left( p \right) } \right) }}{{PVar\left( S \right) }}} \right) , \end{aligned}$$

where $PVar\left( S \right)$ denotes the partial variance of *S*. Assume that the linguistic terms in *S* are equal probability, namely, $S = \left\{ {{s_0},{s_1}, \ldots ,{s_6}} \right\}$, $PVar\left( S \right) \approx 0.1139$.

### Definition 9

Let ${L_1}\left( p \right)$ and ${L_2}\left( p \right)$ be two PLTSs, and the comparison rules are expressed as follows:

1. If $$SF\left( {{L_1}\left( p \right) } \right) $$ > $$SF\left( {{L_2}\left( p \right) } \right) $$, then $${L_1}\left( p \right) $$ > $${L_2}\left( p \right) $$;
2. If $$SF\left( {{L_1}\left( p \right) } \right) $$ = $$SF\left( {{L_2}\left( p \right) } \right) $$, then $${L_1}\left( p \right) $$ = $${L_2}\left( p \right) $$.

## Improved the classic CODAS method

The classic CODAS method was originally proposed by Ghorabaee et al. (2016). As an effective MCDM method, it has a wide range of applications, such as risk assessment, supplier management and so on. In this method, the desirability of alternatives is determined using the Euclidean distance and Hamming distance, which is the farthest from the negative ideal solution. The classic CODAS method directly adds Euclidean distances and Hamming distances under each criterion without considering the influence of criteria weights, which is inconsistent with the actual situation. Therefore, in this part, we work on improving the classic CODAS method by introducing criteria weights information, and extend it to the probabilistic linguistic environment.

### Deficiencies of classic CODAS method

**Fig. 1:**
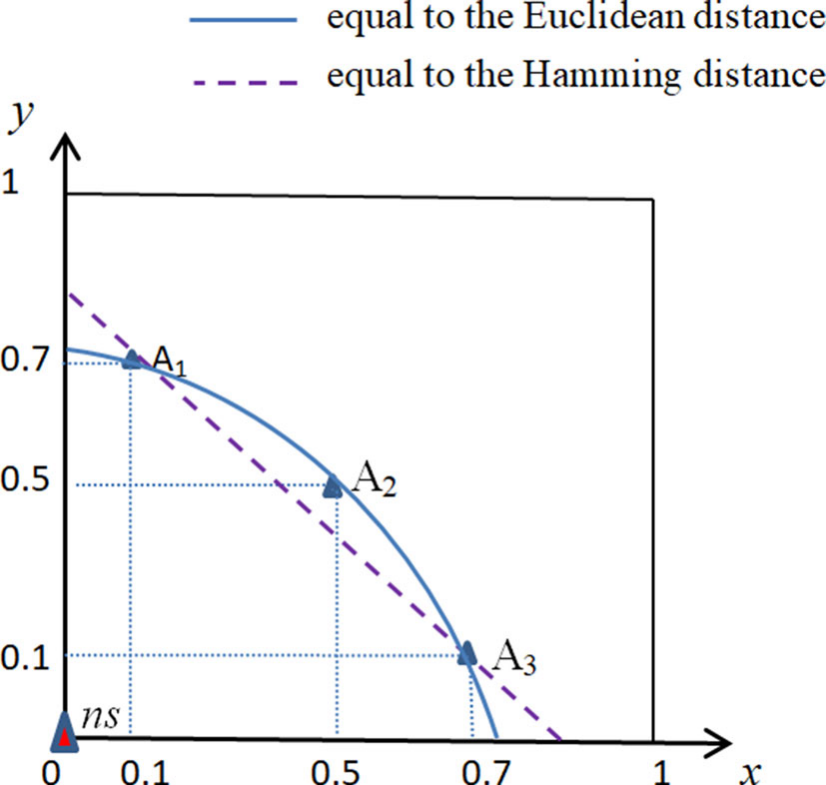
A simple graphical example with two criteria

In view of describe the shortcomings of classic methods, we use a simple situation with three alternatives and two criteria to illustrate. These values are dimensionless and between 0 and 1. Figure 1 shows the position of all alternatives according to these values.

Here, we consider the origin to be the negative ideal solution. The points in the figure are expressed as $ns = \left( {0,0} \right)$, ${A_1} = \left( {0.1,0.9} \right)$, ${A_2} = \left( {0.5,0.75498} \right)$, ${A_3} = \left( {0.9,0.1} \right)$. The Euclidean distances of alternatives from origin are

$$\begin{aligned} {E_1}= & {} \sqrt{{{0.1}^2} + {{0.7}^2}} = \sqrt{0.5}, \\{E_2}= & {} \sqrt{{{0.5}^2} + {{0.5}^2}} = \sqrt{0.5}, \\{E_3}= & {} \sqrt{{{0.7}^2} + {{0.1}^2}} = \sqrt{0.5}. \end{aligned}$$

The Hamming distances of alternatives from origin are

$$\begin{aligned} {H_1}= & {} \left| {0.1} \right| + \left| {0.7} \right| = 0.8,\\{H_2}= & {} \left| {0.5} \right| + \left| {0.5} \right| = 1,\\{H_3}= & {} \left| {0.7} \right| + \left| {0.1} \right| = 0.8. \end{aligned}$$

From the above calculation, we can find that ${A_1}$ is better than ${A_2}$ and equal to ${A_3}$ by the classic CODAS methods. It is easy to check that ${A_1}$, ${A_2}$ and ${A_3}$ are the different alternatives, but the above methods cannot distinguish ${A_1}$ and ${A_3}$. To make up the deficiencies of the classic methods, in the following improved method, we consider the weighted Hamming distance instead of the Hamming distance that was directly added.

### An improved description of the classic CODAS method

Because of the shortcomings of the CODAS method proposed in Sect. 4.1, we improve the classic CODAS method in this section.

1. To overcome the shortcomings of the classic CODAS method described in Sect. 4.1, we use the weighted Hamming distance structure instead of the Hamming distance, and then construct the relative evaluation matrix in this section.
2. In the classic CODAS method, the valve function for constructing the relative evaluation matrix is expressed as follows:
  13 $$\begin{aligned} \psi \left( x \right) = \left\{ {\begin{array}{*{20}{c}} {\begin{array}{*{20}{l}} {0,}&{}\quad {\left| x \right| < \delta ;} \end{array}}\\ {\begin{array}{*{20}{l}} {1,}&{}\quad {\left| x \right| \ge \delta .} \end{array}} \end{array}} \right. \end{aligned}$$

Here, the valve function takes 0 to indicate that the relative evaluation value is the difference between the Euclidean distances of the two alternatives, and the valve function takes 1 to indicate the relative evaluation value is the sum of the difference between the Euclidean distances of the two alternatives and the Hamming distance. Therefore, the meaning of the above function is as follows:if the Euclidean distance difference *x* between the two alternatives is less than the given threshold, the relative evaluation value is the Euclidean distance difference. If the difference *x* between the Euclidean distance of the two alternatives is greater than or equal to the given threshold, the relative evaluation value is the sum of the difference between the Euclidean distance of the two alternatives and the difference of the Hamming distance.

However, the description here is inconsistent with realistic thinking. In general, when the difference *x* between the Euclidean distances of the two alternatives is less than the given threshold, it is considered that only considering the difference between the Euclidean distances is not enough to distinguish the two alternatives. So the Hamming distances needs to be added to distinguish the two alternatives. When the difference *x* between the Euclidean distances of the two alternatives is greater than or equal to the given threshold, the Euclidean distance is considered to be sufficient to distinguish the two alternatives, and Hamming distance does not need to be considered. Based on the above description, we made the following improvements to the definition of valve function.

14 $$\begin{aligned} \psi \left( x \right) = \left\{ {\begin{array}{*{20}{c}} {\begin{array}{*{20}{l}} {0,}&{}\quad {\left| x \right| \ge \delta ;} \end{array}}\\ {\begin{array}{*{20}{l}} {1,}&{}\quad {\left| x \right| < \delta .} \end{array}} \end{array}} \right. \end{aligned}$$

## PL-CODAS method

In this section, we introduce PL-CODAS method. First of all, we determine the criteria weights on the basis of the existing weight decision method. Then based on the improved CODAS method, we extended it to the probabilistic linguistic environment and propose PL-CODAS method for handling the MCDM problems.

### Determine the criteria weights of PLTS

Determining the criterion weights is critical in the whole decision-making process, which can directly affect the outcome of the decision. In many cases, criteria weights are given directly by DMs and it will result in a more subjective outcome. To make up for this deficiency and get more reliable results, refer to Zhang et al. (2019) for the weight calculation method, which consider not only the deviations of all the alternatives to the positive ideal solution, but also the information entropy of all alternatives. In this article, we also utilize this method to determine the weights of criteria.

A given standardized decision matrix $D = {\left( {{L_{ij}}\left( p \right) } \right) _{m \times n}}$, ${L_{ij}}\left( p \right) = \left\{ {L_{ij}^{\left( k \right) }\left( {p_{ij}^{\left( k \right) }} \right) \left| {k = 1,2, \ldots ,\# {L_{ij}}\left( p \right) } \right. } \right\}$

$\left( {i = 1,2, \ldots ,m;j = 1,2, \ldots ,n} \right)$. ${L_{ij}}\left( p \right)$ denotes the performance value of *i*th alternative with respect to *j*th criterion, which is a probabilistic linguistic term set form and has been standardized by Definition 5. The steps to calculate criteria weights are as follows:

**Step 1**  Transform the PLTS into crisp numbers and obtain new matrix $B = {\left( {{b_{ij}}} \right) _{m \times n}}$.

15 $$\begin{aligned} {b_{ij}} = \frac{1}{{\# {L_{ij}}\left( p \right) }}\sum \nolimits _{k = 1}^\eta {b_{ij}^{(k)}}, \end{aligned}$$

where $b_{ij}^{\left( k \right) } = \frac{{r_{ij}^{\left( k \right) }p_{ij}^{\left( k \right) } - \mathop {\min }\limits _i \left( {r_{ij}^{\left( k \right) }p_{ij}^{\left( k \right) }} \right) }}{{\mathop {\max }\limits _i \left( {r_{ij}^{\left( k \right) }p_{ij}^{\left( k \right) }} \right) - \mathop {\min }\limits _i \left( {r_{ij}^{\left( k \right) }p_{ij}^{\left( k \right) }} \right) }}$, if $\mathop {\max }\limits _i \left( {r_{ij}^{\left( k \right) }p_{ij}^{\left( k \right) }} \right) = \mathop {\min }\limits _i \left( {r_{ij}^{\left( k \right) }p_{ij}^{\left( k \right) }} \right)$, then $b_{ij}^{\left( k \right) } = 1$. ${\# {L_{ij}}\left( p \right) }$ denotes the number of PLEs contained in ${L_{ij}}\left( p \right)$, ${r_{ij}^{\left( k \right) }}$ is the subscript of the linguistic term ${L_{ij}^{\left( k \right) }}$.

**Step 2**  Based on the idea of minimizing the deviation from the positive ideal solution and maximizing the uncertainty of the criteria weights, then we construct the following single-objective optimization model to determine the weights of criteria.

16 $$\begin{aligned} \begin{array}{l} \min \quad \left\{ {\mu \sum \limits _{j = 1}^n {{w_j}\sqrt{\sum \limits _{i = 1}^m {{{\left( {b_j^* - {b_{ij}}} \right) }^2}} } + \left( {1 - \mu } \right) {w_j}\ln {w_j}} } \right\} ,\\ s.t.\quad {w_j} \ge 0,j = 1,2, \ldots ,n,\sum \nolimits _{j = 1}^n {{w_j}} = 1, \end{array} \end{aligned}$$

where $$b_j^* $$ = $$\mathop {\max }\limits _i {b_{ij}}$$, $$\mu $$ is a balance coefficient between two objectives satisfies $$0< \mu < 1$$, and can be determined according to the practical problems. Without lose generality, we set $$\mu $$=0.5.

**Step 3**  Solve the optimization model and the weight of each criterion is obtained as

17 $$\begin{aligned} {w_j} = \frac{{\exp \left( { - \frac{\mu }{{1 - \mu }}\sqrt{\sum \nolimits _{i = 1}^m {{{\left( {b_j^* - {b_{ij}}} \right) }^2}} } - 1} \right) }}{{\sum \nolimits _{j = 1}^n {\exp \left( { - \frac{\mu }{{1 - \mu }}\sqrt{\sum \nolimits _{i = 1}^m {{{\left( {b_j^* - {b_{ij}}} \right) }^2}} } - 1} \right) } }},\quad j = 1,2, \ldots ,n. \end{aligned}$$

### Procedures of probabilistic linguistic CODAS method

In the following, we consider the MCDM problem with probabilistic linguistic information. Let $X = \left\{ {{x_1},{x_2}, \ldots ,{x_m}} \right\}$ be a finite set of *m* alternatives, and $C = \left\{ {{c_1},{c_2}, \ldots ,{c_n}} \right\}$ be a finite set of *n* criteria. The specific steps of probabilistic linguistic CODAS method are shown as follows:

**Step 1**  Construct the probabilistic linguistic decision matrix *D*, which is shown as follows:

$$\begin{aligned} D = {\left( {{L_{ij}}\left( p \right) } \right) _{m \times n}} = \left( {\begin{array}{*{20}{c}} {{L_{11}}\left( p \right) }&{}{{L_{12}}\left( p \right) }&{} \cdots &{}{{L_{1n}}\left( p \right) }\\ {{L_{21}}\left( p \right) }&{}{{L_{22}}\left( p \right) }&{} \cdots &{}{{L_{2n}}\left( p \right) }\\ \vdots &{} \vdots &{} \vdots &{} \vdots \\ {{L_{m1}}\left( p \right) }&{}{{L_{m2}}\left( p \right) }&{} \cdots &{}{{L_{mn}}\left( p \right) } \end{array}} \right) , \end{aligned}$$

where ${L_{ij}}\left( p \right) = \left\{ {L_{ij}^{\left( k \right) }\left( {p_{ij}^{\left( k \right) }} \right) \left| {k = 1,2, \ldots ,\# {L_{ij}}\left( p \right) } \right. } \right\}$, which denotes the performance value of *i*th alternative with respect to *j*th criterion$\left( {i = 1,2, \ldots ,m;j = 1,2, \ldots ,n} \right)$.

**Step 2**  Unify the criteria type to get the decision matrix ${\tilde{D}} = {\left( {{{\tilde{L}}_{ij}}\left( p \right) } \right) _{m \times n}}$. The criteria are usually divided into benefit type and cost type, and the larger the value of the benefit type criterion the better, and the smaller the value of the cost type criterion the better. To facilitate the calculation, the common method is to uniformly convert the criteria to the benefit type. The conversion method is as follows:

18 $$\begin{aligned} {{\tilde{L}}_{ij}}\left( p \right) = \left\{ {\begin{array}{*{20}{l}} {{L_{ij}}\left( p \right) ,}&{}\quad {\mathrm{{if\,\,criteria\,\,j\,\,is\,\,benefit\,\,type;}}}\\ {neg\left( {{L_{ij}}\left( p \right) } \right) ,}&{}\quad {\mathrm{{if\,\,criteria\,\,j\,\,is\,\,cost\,\,type}}\mathrm{{.}}} \end{array}} \right. \end{aligned}$$

**Step 3**  Normalize the decision matrix ${\tilde{D}} = {\left( {{{{\tilde{L}}}_{ij}}\left( p \right) } \right) _{m \times n}}$ into ${\bar{D}} = {\left( {{{{\bar{L}}}_{ij}}\left( p \right) } \right) _{m \times n}}$ by Definition 5, where ${{\bar{L}}_{ij}}\left( p \right) = \left\{ {{\bar{L}}_{ij}^{\left( k \right) }\left( {{\bar{p}}_{ij}^{\left( k \right) }} \right) \left| {k = 1,2, \ldots ,\# {L_{ij}}\left( p \right) } \right. } \right\}$.

**Step 4**  Determine the negative ideal alternative under each criterion as follows:

19 $$\begin{aligned} ns= & {} {\left( {{x_{{t_j}}}} \right) _{1 \times n}}, \end{aligned}$$

20 $$\begin{aligned} {t_j}= & {} indx\left( {\mathop {\min }\limits _i \left\{ {SF\left( {{{{\bar{L}}}_{ij}}\left( p \right) } \right) } \right\} } \right) , \end{aligned}$$

where ${SF\left( {{{{\bar{L}}}_{ij}}\left( p \right) } \right) }$ denotes the score value of ${{{{\bar{L}}}_{ij}}\left( p \right) }$ and can be obtained by Eq. (12), and $indx\left( {\mathop {\min }\limits _i \left\{ {SF\left( {{{{\bar{L}}}_{ij}}\left( p \right) } \right) } \right\} } \right)$ represents the index value of the alternative with the smallest score function under criterion *j*.

To better understand the calculation process of the negative ideal solution under each criterion, an example is given below for illustration.

#### Example 1

Suppose the evaluation values of the four alternative under criterion 1 are ${L_{11}} = \left\{ {{s_{\mathrm{{4}}}}(0.6),{s_5}(0.3),} \right.$ $\left. {{s_3}(0.1),{s_3}(0)} \right\}$, ${L_{21}} = \left\{ {\left. {{s_{\mathrm{{4}}}}(0.6),{s_5}(0.4),{s_4}(0),{s_4}(0)} \right\} } \right.$, ${L_{31}} = \left\{ {\left. {{s_2}(0.8),{s_1}(0.2),{s_1}(0),{s_1}(0)} \right\} } \right.$ and ${L_{41}} = \left\{ {{s_5}(0.5),} \right.$ $\left. {{s_3}(0.3),{s_4}(0.1),{s_2}(0.1)} \right\}$. The score functions of the four evaluation values calculated from Definitions 7 and 8 are $SF\left( {{L_{11}}} \right) = 0.628$, $SF\left( {{L_{21}}} \right) = 0.657$, $SF\left( {{L_{31}}} \right) = 0.299$ and $SF\left( {{L_{41}}} \right) = 0.499$. Therefore, the score of alternative 3 is the lowest under criterion 1. In other words, the negative ideal solution under criterion 1 is $\left\{ {\left. {{s_2}(0.8),{s_1}(0.2),{s_1}(0),{s_1}(0)} \right\} } \right.$.

**Step 5**  Calculate the weight of each criterion by Eqs. (15)–(17).

**Step 6**  Compute the weighted normalized decision matrix *R*.

21 $$\begin{aligned} {r_{ij}}\left( p \right) = {w_j}{{\bar{L}}_{ij}}\left( p \right) , \end{aligned}$$

where ${w_j}{{\bar{L}}_{ij}}\left( p \right)$ is calculated by operation (3) of Definition 3.

**Step 7**  Calculate the Euclidean and Hamming distances of alternatives from the negative ideal alternatives, shown as follows:

22 $$\begin{aligned} {E_i}= & {} \sqrt{\sum \nolimits _{j = 1}^n {{d_{ne}}{{\left( {{r_{ij}}\left( p \right) ,{r_{{t_j}j}}\left( p \right) } \right) }^2}} }, \end{aligned}$$

23 $$\begin{aligned} {H_i}= & {} \sum \nolimits _{j = 1}^n {{w_j}{d_{nh}}\left( {{r_{ij}}\left( p \right) ,{r_{{t_j}j}}\left( p \right) } \right) }, \end{aligned}$$

where ${{d_{nh}}\left( {{r_{ij}}\left( p \right) ,{r_{{t_j}j}}\left( p \right) } \right) }$ and ${{d_{ne}}\left( {{r_{ij}}\left( p \right) ,{r_{{t_j}j}}\left( p \right) } \right) }$ are calculated by Eqs. (7) and (8), respectively.

**Step 8**  Construct the relevant evaluation matrix *Q*, shown as follows:

24 $$\begin{aligned} Q= & {} {\left( {{q_{ik}}} \right) _{m \times m}}, \end{aligned}$$

25 $$\begin{aligned} {q_{ik}}= & {} \left( {{E_i} - {E_k}} \right) + \left( {\psi \left( {{E_i} - {E_k}} \right) \times \left( {{H_i} - {H_k}} \right) } \right) . \end{aligned}$$

where $\left( {k = 1,2, \ldots ,m} \right)$ and $\psi$ denotes a threshold function to recognize the equality of the Euclidean distances of two alternatives, and is defined as follows:

26 $$\begin{aligned} \psi \left( {{E_i} - {E_k}} \right) = \left\{ {\begin{array}{*{20}{c}} {\begin{array}{*{20}{c}} {0,}&{}\quad {\left| {{E_i} - {E_k}} \right| \ge \delta ;} \end{array}}\\ {\begin{array}{*{20}{c}} {1,}&{}\quad {\left| {{E_i} - {E_k}} \right| < \delta .} \end{array}} \end{array}} \right. \end{aligned}$$

**Step 9**  Calculate the comprehensive evaluation value of each alternative shown as follows:

27 $$\begin{aligned} {q_i} = \sum \nolimits _{k = 1}^m {{q_{ik}}}. \end{aligned}$$

**Step 10**  Sort the alternatives by the comprehensive evaluation values $$q_i $$. The alternative with the highest $$q_i $$ is the best choice among the alternatives.

**Fig. 2:**
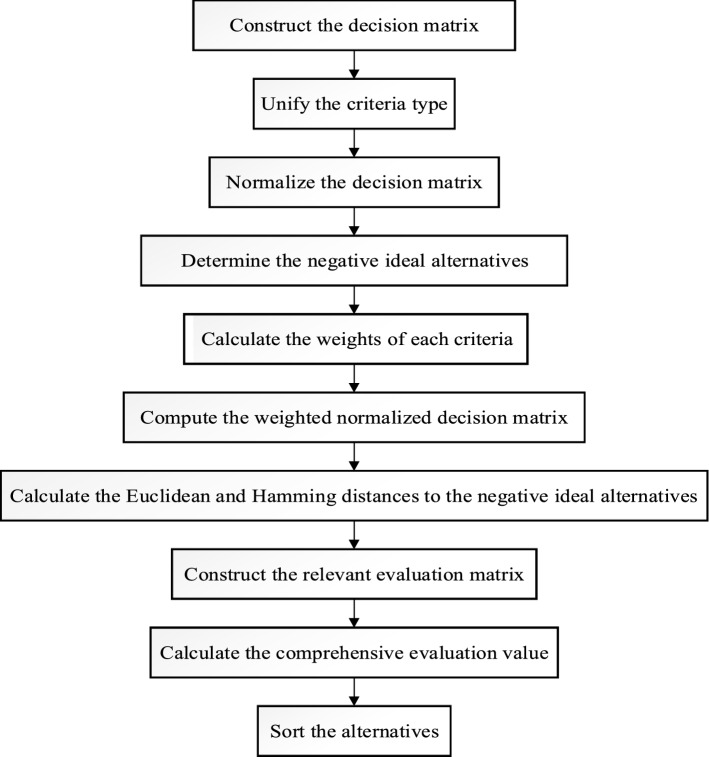
Flowchart of the PL-CODAS method

To better understand the steps of PL-CODAS method, we draw its flowchart as shown in Figure 2.

## Case analysis

In this section, we use an example (adapted from Zhou and Xu 2019) to illustrate the effectiveness and practicality of the proposed PL-CODAS method.

### Problem description

A risk investor plans to place an idle fund of $\$$1,000,000 in the Growth Enterprises Market board of the Shenzhen Stock Exchange in China to obtain high returns which are accompanied by high risks. This investor considers four new companies $x_i$ $\left( {i = 1,1,3,4} \right)$ in the Growth Enterprises Market board that are listed in 2018. They are SangFor Technologies $x_1$(SFT, code: 300454), Sunshine Global Circuits $x_2$(SGC, code: 300739), Tianyi Comheart Telecom $x_3$ (TCT, code: 300504), and Contemporary Amperex Technology $x_4$(CAT, code: 300750). These companies represent four emerging and potential industries, which were unstable and even unprofitable in the latest two years according to their listing reports. Financial experts and managers were invited to conduct a qualitative assessment of the development potential and future earnings of the four companies based on the following three main indicators: technical feasibility $c_1$, future earnings $c_2$, and core team $c_3$, where three indicators are benefit indicators. Let $S = \left\{ {{s_0},{s_1}, \ldots ,{s_6}} \right\}$ = $\left\{ {very \;\;poor, poor, slightly \;\;poor, fair, slightly \;\;good, good, very \;\;good} \right\}$ be the set of evaluation LTS, and the invited expert utilize PLTSs to express his/her evaluation for four new companies with respect to the three main criteria. We solve this problem using the procedure mentioned in Sect. 5.2.

### The evaluation steps

In the following, the steps of using the proposed PL-CODAS method for the evaluation of risk investment are presented:

**Step 1**  Construct the probabilistic linguistic decision matrix $D = {\left( {{L_{ij}}\left( p \right) } \right) _{4 \times 3}}$($i = 1,2,3,4;j = 1,2,3$) shown in Table 1.

**Table 1:** The decision matrix *D*

	$c_1$	$c_2$	$c_3$
$x_1$	$\left\{ {\left. {{s_{\mathrm{{5}}}}(0.\mathrm{{3}}),{s_{\mathrm{{4}}}}(0.\mathrm{{6}}),{s_{\mathrm{{3}}}}(0.1)} \right\} } \right.$	$\left\{ {\left. {{s_{\mathrm{{5}}}}(0.\mathrm{{8}}),{s_{\mathrm{{4}}}}(0.\mathrm{{2}})} \right\} } \right.$	$\left\{ {\left. {{s_{\mathrm{{4}}}}(0.\mathrm{{7}}),{s_{\mathrm{{3}}}}(0.\mathrm{{1}}),{s_{\mathrm{{2}}}}(0.1),{s_1}(0.1)} \right\} } \right.$
$x_2$	$\left\{ {\left. {{s_{\mathrm{{5}}}}(0.4),{s_{\mathrm{{4}}}}(0.6)} \right\} } \right.$	$\left\{ {\left. {{s_3}(0.\mathrm{{8}}),{s_2}(0.\mathrm{{2}})} \right\} } \right.$	$\left\{ {\left. {{s_{\mathrm{{5}}}}(0.7),{s_{\mathrm{{4}}}}(0.2),{s_{\mathrm{{3}}}}(0.1)} \right\} } \right.$
$x_3$	$\left\{ {\left. {{s_2}(0.8),{s_1}(0.2)} \right\} } \right.$	$\left\{ {\left. {{s_5}(0.6),{s_4}(0.\mathrm{{1}}),{s_3}(0.2),{s_2}(0.1)} \right\} } \right.$	$\left\{ {\left. {{s_{\mathrm{{5}}}}(0.7),{s_{\mathrm{{4}}}}(0.3)} \right\} } \right.$
$x_4$	$\left\{ {\left. {{s_5}(0.5),{s_4}(0.\mathrm{{1}}),{s_3}(0.2),{s_2}(0.1)} \right\} } \right.$	$\left\{ {\left. {{s_{\mathrm{{5}}}}(0.6),{s_3}(0.3),{s_{\mathrm{{2}}}}(0.1)} \right\} } \right.$	$\left\{ {\left. {{s_5}(0.3),{s_4}(0.7)} \right\} } \right.$

**Step 2**  In this example, all criteria are benefit type, and the decision matrix after the unified the criteria type is still Table 1.

**Step 3**  Normalize the decision matrix $D = {\left( {{L_{ij}}\left( p \right) } \right) _{4 \times 3}}$ into $\bar{D} = {\left( {{{{\bar{L}}}_{ij}}\left( p \right) } \right) _{4 \times 3}}$ according to Definitions 4 and 5, shown in Table 2.

**Table 2:** The normalized decision matrix ${{\bar{D}}}$

	$c_1$	$c_2$	$c_3$
$x_1$	$\left\{ {\left. {{s_{\mathrm{{4}}}}(0.6),{s_5}(0.3),{s_3}(0.1),{s_3}(0)} \right\} } \right.$	$\left\{ {\left. {{s_5}(0.8),{s_4}(0.2),{s_4}(0),{s_4}(0)} \right\} } \right.$	$\left\{ {\left. {{s_{\mathrm{{4}}}}(0.7),{s_3}(0.1),{s_2}(0.1),{s_1}(0.1)} \right\} } \right.$
$x_2$	$\left\{ {\left. {{s_{\mathrm{{4}}}}(0.6),{s_5}(0.4),{s_4}(0),{s_4}(0)} \right\} } \right.$	$\left\{ {\left. {{s_3}(0.8),{s_2}(0.2),{s_2}(0),{s_2}(0)} \right\} } \right.$	$\left\{ {\left. {{s_5}(0.7),{s_4}(0.2),{s_3}(0.1),{s_3}(0)} \right\} } \right.$
$x_3$	$\left\{ {\left. {{s_2}(0.8),{s_1}(0.2),{s_1}(0),{s_1}(0)} \right\} } \right.$	$\left\{ {\left. {{s_5}(0.6),{s_3}(0.2),{s_4}(0.1),{s_2}(0.1)} \right\} } \right.$	$\left\{ {\left. {{s_5}(0.7),{s_4}(0.3),{s_4}(0),{s_4}(0)} \right\} } \right.$
$x_4$	$\left\{ {\left. {{s_5}(0.5),{s_3}(0.3),{s_4}(0.1),{s_2}(0.1)} \right\} } \right.$	$\left\{ {\left. {{s_5}(0.6),{s_3}(0.3),{s_2}(0.1),{s_2}(0)} \right\} } \right.$	$\left\{ {\left. {{s_4}(0.7),{s_3}(0.3),{s_3}(0),{s_3}(0)} \right\} } \right.$

**Step 4**  Determine the negative ideal solution.

According to Table 2, the scores of the four companies under the three main criteria are calculated by the calculation method proposed in Sect. 4 shown in Table 3.

**Table 3:** Score value of four hospitals under three criteria

	$c_1$	$c_2$	$c_3$
$x_1$	0.628	0.638	0.335
$x_2$	0.657	0.439	0.575
$x_3$	0.299	0.463	0.649
$x_4$	0.499	0.444	0.548

From Table 3, the score of $x_3$ is the lowest under technical feasibility. $x_2$ has the lowest score under future earnings. Under core team, $x_1$ has the lowest score. The negative ideal hospitals under three criterion is $( \left\{ {{s_2}(0.8),{s_1}(0.2),{s_1}(0),{s_1}(0)} \right\} ,\left\{ {{s_3}(0.8),{s_2}(0.2),{s_2}(0),{s_2}(0)} \right\}$, $\left\{ {s_4}(0.7),{s_3}(0.1),{s_2}(0.1),{s_1}(0.1) \right\} )$.

**Step 5**  Calculate criteria weights. Transform decision matrix *D* into crisp number matrix *B* by Eq. (15).

$$\begin{aligned} B = \left( {\begin{array}{*{20}{c}} {0.590}&{}\quad {0.450}&{}\quad {0.417}\\ {0.472}&{}\quad 0&{}\quad {0.639}\\ 0&{}\quad {0.694}&{}\quad {0.500}\\ {0.847}&{}\quad {0.469}&{}\quad {0.167}. \end{array}} \right) \end{aligned}$$

Through crisp number matrix *B*, the criteria weights is 0.268,0.324,0.408 by Eqs. (16) and (17).

**Step 6**  Calculate the weighted normalized decision matrix *R* by Eq. (21), shown in Table 4.

**Table 4:** The weighted normalized decision matrix

	$c_1$	$c_2$	$c_3$
$x_1$	$\left\{ {\left. {{s_{1.03}}(0.6),{s_{1.66}}(0.3),{s_{0.74}}(0.1),{s_{0.74}}(0)} \right\} } \right.$	$\left\{ {\left. {{s_{2.04}}(0.8),{s_{1.26}}(0.2),{s_{1.26}}(0),{s_{1.26}}(0)} \right\} } \right.$	$\left\{ {\left. {{s_{1.6}}(0.7),{s_{1.14}}(0.1),{s_{0.78}}(0.1),{s_{0.4}}(0.1)} \right\} } \right.$
$x_2$	$\left\{ {\left. {{s_{1.03}}(0.6),{s_{1.66}}(0.4),{s_{1.03}}(0),{s_{1.03}}(0)} \right\} } \right.$	$\left\{ {\left. {{s_{0.9}}(0.8),{s_{0.62}}(0.2),{s_{0.62}}(0),{s_{0.62}}(0)} \right\} } \right.$	$\left\{ {\left. {{s_{2.62}}(0.7),{s_{1.6}}(0.2),{s_{1.6}}(0.1),{s_{1.6}}(0)} \right\} } \right.$
$x_3$	$\left\{ {\left. {{s_{0.51}}(0.8),{s_{0.26}}(0.2),{s_{0.26}}(0),{s_{0.26}}(0)} \right\} } \right.$	$\left\{ {\left. {{s_{2.04}}(0.6),{s_{0.62}}(0.2),{s_{1.26}}(0.1),{s_{0.62}}(0.1)} \right\} } \right.$	$\left\{ {\left. {{s_{2.62}}(0.7),{s_{1.6}}(0.3),{s_{1.6}}(0),{s_{1.6}}(0)} \right\} } \right.$
$x_4$	$\left\{ {\left. {{s_{1.66}}(0.5),{s_{0.74}}(0.3),{s_{1.03}}(0.1),{s_{0.51}}(0.1)} \right\} } \right.$	$\left\{ {\left. {{s_{2.04}}(0.6),{s_{0.9}}(0.3),{s_{0.62}}(0.1),{s_{0.62}}(0)} \right\} } \right.$	$\left\{ {\left. {{s_{1.62}}(0.7),{s_{1.14}}(0.3),{s_{1.14}}(0),{s_{1.14}}(0)} \right\} } \right.$

**Step 7**  Calculate the Euclidean and Hamming distances.

According to the matrix *R*, under each criterion, the Hamming distance and the Euclidean distance between the four companies and the worst company are calculated as Tables 5 and 6 by Eqs. (7) and (8), respectively.

**Table 5:** Hamming distance between the four companies and the worst company

	$c_1$	$c_2$	$c_3$
$x_1$	0.036	0.043	0
$x_2$	0.040	0	0.038
$x_3$	0	0.031	0.049
$x_4$	0.360	0.029	0.019

**Table 6:** Euclidean distance between the four companies and the worst company

	$c_1$	$c_2$	$c_3$
$x_1$	0.048	0.075	0
$x_2$	0.063	0	0.052
$x_3$	0	0.039	0.061
$x_4$	0.042	0.040	0.027

According to Tables 5 and  6, the weighted Hamming distance and Euclidean distance between the four companies and the negative ideal company are calculated as *H* and *E* by Eqs. (22) and (23), respectively.

$$\begin{aligned} H = {(\begin{array}{*{20}{c}} {0.024}&{0.026}&{0.030}&{0.027} \end{array})^T}\\E = {(\begin{array}{*{20}{c}} {0.090}&{0.082}&{0.072}&{0.063} \end{array})^T}. \end{aligned}$$

**Step 8**  Construct the relevant evaluation matrix *Q* by Eqs. (24)–(26).

$$\begin{aligned} Q = {({q_{ik}})_{4 \times 4}} = \left( {\begin{array}{*{20}{c}} 0&{}\quad {0.008}&{}\quad {0.017}&{}\quad {0.023}\\ { - 0.010}&{}\quad 0&{}\quad {0.010}&{}\quad {0.018}\\ { - 0.020}&{}\quad { - 0.010}&{}\quad 0&{}\quad {0.090}\\ { - 0.020}&{}\quad { - 0.020}&{}\quad { - 0.010}&{}\quad 0 \end{array}} \right) . \end{aligned}$$

**Step 9**   Calculate the comprehensive evaluation value $$Q_i $$ of each company by Eq. (27).

$$\begin{aligned} {Q_1} = 0.0484,{Q_2} = 0.0201,{Q_3} = -0.018,{Q_4} = - 0.05. \end{aligned}$$

**Step 10**  Sort the companies by the comprehensive evaluation values $$Q_i $$.

$$\begin{aligned} {x_1} \succ {x_2} \succ {x_3} \succ {x_4}. \end{aligned}$$

Thus, the the most suitable company for investment is $$x_2 $$, i.e., Sangfor Technologies company. Once the values of threshold parameter change, the decision results may change as well. The decision outcomes with different values of $$\delta $$ are shown in Table 7.

**Table 7:** The comprehensive evaluation of four companies

	Parameter	$x_1$	$x_2$	$x_3$	$x_4$
Comprehensive evaluation	$\delta = 0.01$	0.0421	0.0194	$-\,0.012$	$-\,0.05$
	$\delta = 0.02$	0.0484	0.0201	$-\,0.018$	$-\,0.05$
	$\delta = 0.05$	0.0516	0.0201	$-\,0.018$	$-\,0.053$

It can be seen from Table 7 that the comprehensive evaluation value for different values of $\delta$ schemes has changed but the ranking of the schemes remains unchanged.

### Comparative analysis

In this section, we demonstrate the rationality and effectiveness of this method by comparing with other existing methods. So far, there is no research on the CODAS method in the probabilistic linguistic environment. Therefore, we use probabilistic linguistic information to compare existing methods for MCDM problems. In the same context, we use existing methods to handle the same case, i.e., PL-TOPSIS method (Lu et al. 2019), PL-TODIM method (Liu and You 2017) and PL-MABAC method (Wei et al. 2019).

#### Comparison with the PL-TOPSIS method

The data in this paper are applied to the PL-TOPSIS method proposed by Lu et al. (2019).

**Steps 1–2**  Similar to steps 1–3 in Sect. 6.2, the results are omitted here.

**Step 3**  Compute the weight values for criteria ${w_j}$, and get ${w_1} = 0.643$, ${w_2} = 0.233$ and ${w_3} = 0.124$.

**Step 4**  Determine the PLPIS and PLNIS as shown in Table 8.

**Table 8:** The PLPIS and PLNIS

	$c_1$	$c_2$	$c_3$
*PLPIS*	$\left\{ {\left. {{s_1}(0),{s_1}(0),{s_1}(0.6),{s_2}(0.4)} \right\} } \right.$	$\left\{ {\left. {{s_1}(0),{s_1}(0),{s_1}(0.2),{s_2}(0.8)} \right\} } \right.$	$\left\{ {\left. {{s_1}(0 ),{s_1}(0 ),{s_1}(0.3),{s_2}(0.7)} \right\} } \right.$
*PINIS*	$\left\{ {\left. {{s_-2}(0 ),{s_-2}(0 ),{s_4}(0),{s_-2}(0.2)} \right\} } \right.$	$\left\{ {\left. {{s_-1}(0 ),{s_-1}(0 ),{s_-1}(0.2),{s_0}(0.8)} \right\} } \right.$	$\left\{ {\left. {{s_-2}(0.1),{s_-1}(0.1),{s_0}(0.1),{s_1}(0.7)} \right\} } \right.$

**Step 5**  Calculate the distances $d\left( {PL{x_i},PLPIS} \right)$ and $d\left( {PL{x_i},PLNIS} \right)$ of each alternative, then we have

$$d\left( {PL{x_1},PLNIS} \right) = 0.089,d\left( {PL{x_2},PLNIS} \right) = 0.077,d\left( {PL{x_3},PLNIS} \right) = 0.023,d\left( {PL{x_4},PLNIS} \right) = 0.076;$$

$$d\left( {PL{x_1},PLPIS} \right) = 0.031,d\left( {PL{x_2},PLPIS} \right) = 0.023,d\left( {PL{x_3},PLPIS} \right) = 0.091,d\left( {PL{x_4},PLPIS} \right) = 0.114.$$

**Step 6**  Calculating the $\mathrm{{PLRCD}}\left( {PL{x_i},PLPIS} \right)$, and get

$$\begin{aligned}&\mathrm{{PLRCD}}\left( {PL{x_1},PLPIS} \right) = 0.74, \mathrm{{PLRCD}}\left( {PL{x_2},PLPIS} \right) = 0.769, \\&\mathrm{{PLRCD}}\left( {PL{x_3},PLPIS} \right) = 0.205 and \mathrm{{PLRCD}}\left( {PL{x_4},PLPIS} \right) = 0.4. \end{aligned}$$

**Step 7**  Rank the alternatives ${{x_i}}$ by the values $\mathrm{{PLRCD}}\left( {PL{x_i},PLPIS} \right)$ $\left( {i = 1,2,3,4} \right)$, and obtain ${x_2} \succ {x_1} \succ {x_4} \succ {x_3}$.

#### Comparison with the PL-TODIM method

The data in this paper are applied to PL-TODIM method proposed by Liu and You (2017).

**Step 1**  Similar to steps 1–3 in Sect. 6.2, the results are omitted here.

**Step 2**  Calculate the criteria weights ${w_j}$ $(j = 1,2,3)$, and we can get

$$\begin{aligned}&{H_1} = 0.147,{H_2} = - 0.033,{H_3} = - 0.101; \\&{w_1} = 0.286,{w_2} = 0.246,{w_3} = 0.369. \end{aligned}$$

**Step 3**  ${c_3}$ is the reference criterion and the reference weight is ${w_r} = 0.369$. Therefore, the relative weights for all the criteria are ${w_{1r}} = 0.775,{w_{2r}} = 0.938 \,\, and \,\,{w_{3r}} = 1$.

**Step 4**  Calculate the dominance of each alternative ${x_i}$ over each alternative ${x_t}$, and get

$${\phi _1} = \left( {\begin{array}{*{20}{c}} 0&{}\quad { - 1.01}&{}\quad {0.471}&{}\quad {0.304}\\ {0.289}&{}\quad 0&{}\quad {0.53}&{}\quad {0.413}\\ { - 1.65}&{}\quad { - 1.86}&{}\quad 0&{}\quad { - 1.46}\\ { - 1.07}&{}\quad { - 1.44}&{}\quad {0.418}&{}\quad 0 \end{array}} \right) ,$$ $${\phi _2} = \left( {\begin{array}{l@{\quad }l@{\quad }l@{\quad }l} 0&{}{0.534}&{}{0.439}&{}{0.421}\\ { - 1.54}&{}0&{}{ - 1.06}&{}{ - 1.08}\\ { - 1.27}&{}{0.366}&{}0&{}{0.267}\\ { - 1.22}&{}{0.373}&{}{ - 0.77}&{}0 \end{array}} \right) ,$$

$${\phi _3} = \left( {\begin{array}{l@{\quad }l@{\quad }l@{\quad }l} 0&{}{ - 1.09}&{}{ - 1.26}&{}{ - 0.93}\\ {0.401}&{}0&{}{ - 0.82}&{}{0.376}\\ {0.463}&{}{0.304}&{}0&{}{0.375}\\ {0.344}&{}{ - 1.02}&{}{ - 1.02}&{}0 \end{array}} \right) .$$

Then we can get the overall dominance degree between alternatives as follows:

$$\begin{aligned} \phi = \left( {\begin{array}{l@{\quad }l@{\quad }l@{\quad }l} 0&{}{ - 1.56}&{}{ - 0.35}&{}{ - 0.221}\\ { - 0.85}&{}0&{}{ - 1.35}&{}{ - 0.29}\\ { - 2.46}&{}{ - 1.19}&{}0&{}{ - 0.82}\\ { - 1.94}&{}{ - 2.09}&{}{ - 1.37}&{}0 \end{array}} \right) . \end{aligned}$$

**Step 5**  Calculate the ${\delta _i}$ of alternative ${x _i}$, and we have ${\delta _1} = 1,{\delta _2} = 0.884,{\delta _3} = 0.285 \,\,and\,\, {\delta _4} = 0$.

**Step 6**  Rank the alternatives ${{x_i}}$ by the values ${\delta _i}$ $\left( {i = 1,2,3,4} \right)$, and obtain ${x_1} \succ {x_2} \succ {x_3} \succ {x_4}$.

#### Comparison with the PL-MABAC method

The data in this paper are applied to PL-MABAC method proposed by Wei et al. (2019).

**Steps 1–3**  Similar to steps 1–3 in Sect. 6.2, the results are omitted here.

**Step 4**  Compute the combined weight values for the criteria. First, the objective weights of criteria were $o{w_1} = 0.427,o{w_2} = 0.311\,\,and\,\,o{w_3} = 0.261$. From here, suppose that the subjective weights of the criteria were $s{w_1} = 0.3,s{w_2} = 0.3\,\,and\,\,s{w_3} = 0.4$. Finally, the combined weight values for criteria were found to be ${w_1} = 0.393,{w_2} = 0.286\,\,and\,\,{w_3} = 0.321$.

**Step 5**  Solve the *PLBAA* and obtain, and have $PLBA{A_1} = \big \{ {s_{ - 0.79}}(0),{s_{ - 0.55}}(0),{s_{ - 0.17}}(0.291),{s_{976}}(0.468) \big \}$, $PLBA{A_2} = \left\{ {{s_{ - 0.62}}(0),{s_{ - 0.37}}(0),{s_{0.13}}(0.186),{s_{1.401}}(0.693)} \right\}$ and $PLBA{A_3} = \left\{ {{s_{ - 0.55}}(0),{s_{ - 0.09}}(0),{s_{0.464}}(0.206),} \right.$ $\left. {{s_{1.472}}(0.7)} \right\}$.

**Step 6**  Compute the PLHD (probabilistic linguistic Hamming distance) from PLBAA as displayed in Table 9.

**Table 9:** PLHD matrix

Alternatives	$c_1$	$c_2$	$c_3$
$x_1$	0.025	0.014	− 0.006
$x_2$	0.028	− 0.01	0.01
$x_3$	− 0.01	0.007	0.011
$x_4$	0.022	0.006	− 0.002

**Step 7**  Calculate the $PLS{V_i}$ of alternative ${x _i}$, and we have $PLS{V_1} = 0.033,PLS{V_2} = 0.028,PLS{V_3} = 0.004 \,\,and\,\, PLS{V_4} = 0.025$.

**Step 8**  Rank the alternatives ${{x_i}}$ by the values $PLS{V_i}$ $\left( {i = 1,2,3,4} \right)$, and obtain ${x_1} \succ {x_2} \succ {x_4} \succ {x_3}$.

#### Discussion

In this section, we compare the proposed method with the above existing methods. The results are shown in Table 10.

**Table 10:** Comparison of four different methods

Methods	Ranking results	Optimal choice
PL-TOPSIS method (Lu et al. 2019)	${x_2} \succ {x_1} \succ {x_4} \succ {x_3}$	${x_2}$
PL-TODIM method (Liu and You 2017 )	${x_1} \succ {x_2} \succ {x_3} \succ {x_4}$	${x_1}$
PL-MABAC method (Wei et al. 2019)	${x_1} \succ {x_2} \succ {x_4} \succ {x_3}$	${x_1}$
PL-CODAS method	${x_1} \succ {x_2} \succ {x_3} \succ {x_4}$	${x_1}$

From Table 10, we can see that the ranking result calculated by PL-TODIM method are the same as that of the proposed method, while the ranking results calculated by the PL-TOPSIS method and PL-MABAC method are slightly different from those calculated by the proposed method. The same ranking order illustrates that the developed approach is valid and reasonable, and the different ranking results can explain the advantages of the proposed method in this paper. We can explain these results as follows.

The PL-TOPSIS method determines the optimal alternative by calculating the shortest distance to the probabilistic linguistic positive ideal solution (PLPIS) and the longest distance to the probabilistic linguistic negative ideal solution (PLNIS), and considers the influence of the PLPIS and the PLNIS on the selection of the alternatives. However, in the literature (Lu et al. 2019), the comparison of the evaluation values of PLTS is based on the comparison of the mean values of linguistic terms. Only when the mean values are equal, the variance is considered to distinguish. In the third section, we analyzed the shortcomings of this type of score function definition, so in this article we redefine the comparison rules for the evaluation value of PLTS, which also consider the mean and partial variance of linguistic terms. Through analysis, we found that the score function of this paper is considered more comprehensive, which also leads to differences in the ranking results obtained by the PL-TOPSIS method and the proposed method.

Based on the prospect theory, the PL-TODIM method determines the optimal alternative by considering the cognitive behavior of the decision-maker. This method can capture losses and gains in uncertain situations from the perspective of a reference point. Through comparative analysis, it is found that the ranking results are same obtained by the PL-TODIM method and the proposed method, which shows the effectiveness of the proposed method in this paper.

The PL-MABAC method obtains the optimal alternative by calculating the difference between the approximate area of the multi-attribute boundary and the alternative. In the literature (Wei et al. 2019), when calculating the difference between multi-attributive border approximation area and the alternatives, only the Hamming distance is considered. Compared with the PL-MABAC method, the proposed method not only considers the Euclidean distance to the negative ideal alternative, but also further considers the Hamming distance when the Euclidean distance does not meet the accuracy requirements, which also leads to a little differences in the ranking results obtained by the PL-MABAC method and the proposed method.

Based on above analysis, the score function that considers the mean and partial deviation proposed in this paper is more comprehensive when comparing probabilistic language term sets. In addition, considering both Euclidean distance and Hamming distance to calculate the gap between probabilistic linguistic term sets makes the ranking results more accurate. In the above two aspects, the developed method is superior to the existing methods.

## Conclusion

In this paper, we expand the CODAS method and combines it with PLTS information to deal with MCDM problems in uncertain environments. First, we have a brief overview of the definition, operational laws, the normalization and distance measure of PLTSs. Then in consideration of the mean of linguistic terms, partial variance and hesitation of PLTS, we define a new score function. Moreover, we improved the classic CODAS method and proposed the PL-CODAS model. Through a cases of venture investors choosing emerging companies, the effectiveness and rationality of the proposed method are illustrated. The major achievements of this paper are listed below:

1. Defined score function on the probabilistic linguistic term set under risk assessment situation.
2. Improved the classic CODAS method and extended it to PLTS.
3. Proposed the PL-CODAS method and applied to actual cases.
4. Compared and analyzed the PL-CODAS method with the PL-TOPSIS method, the PL-TODIM method and the PL-MABAC method, and demonstrated the effectiveness and applicability of the PL-CODAS method.

It is noteworthy that this study still has some shortcomings that warrant further research. First, in this paper, we directly quotes the existing literature methods when calculating the criteria weights, and further research on the calculation of criteria weights is needed in the future. Moreover, we have only proposed an expert decision-making model and method. In the future, we will apply this method to more practical situations, such as the prediction the number of people infected with COVID-19.
